# Family Income and Child Depression: The Chain Mediating Effect of Parental Involvement, Children’s Self-Esteem, and Group Differences

**DOI:** 10.3390/children11040478

**Published:** 2024-04-16

**Authors:** Xi Quan, Hanning Lei, Chengwei Zhu, Yun Wang, Furong Lu, Cai Zhang

**Affiliations:** 1 Collaborative Innovation Center of Assessment for Basic Education Quality, Beijing Normal University, Beijing 100875, China; 2 State Key Laboratory of Cognitive Neuroscience and Learning, Beijing Normal University, Beijing 100875, China; 3 School of Education Science, Shanxi University, Taiyuan 030006, China

**Keywords:** depression, family income, parental involvement, self-esteem, single-parent family

## Abstract

Family income is an important factor that affects depression in children and can indirectly be associated with children’s development through family and individual factors. However, few studies have examined the mechanism of multiple risk factors. Therefore, this study focused on the relationship between family income and child depression, as well as the chain mediating the roles of parental involvement and children’s self-esteem both in single-parent families and intact families. A total of 1355 primary school students completed questionnaires that assessed family income, parental involvement, children’s self-esteem, and depression. The results showed that family income influenced child depression through both the mediating roles of parental involvement and children’s self-esteem and the chain mediating role of parental involvement and children’s self-esteem. Meanwhile, family income only influenced child depression through chain mediation in single-parent families. The group differences in the mechanism of depression provide a reference for empirical research on depression intervention in children from different family structures.

## 1. Introduction

Depression is a common mental illness [1] that can lead to low mood, cognitive and behavioral impairment, and even suicide in children [2,3]. More and more young children are suffering from depression, and the early onset of depression can pose greater and longer-lasting harm [4]. Therefore, studying the influencing factors and influencing mechanisms of depression in children is helpful for understanding the reasons for the high depressive incidence in youth and to facilitate prevention and intervention. A developmentally informed theoretical integration of a vulnerability and stress model for depression called the elaborated vulnerability–transactional stress model was proposed to account for the development of depression as a complex multifactorial disorder [5], which suggests that the vulnerability and the stressors of depression can interact and lead to depression. The stress factors mainly include the family environment, negative life events, as well as the learning environment, and vulnerability factors mainly refer to attributional styles, personality, coping styles, and self-perceptions [6].

Studies have shown that family factors have a great impact on depression [7], as a single-parent family, a low family income, and negative parenting are important stress factors [8], while self-esteem is an important cognitive vulnerability factor [9]. In addition, prior research has found that stressors often appear together, and multiple stressors have a greater impact on the development of children than a single stressor [10]. However, previous studies either studied stress factors and susceptibility factors separately or discussed a single stress factor and susceptibility factor [11,12,13]. This study aimed to explore the combined effects of multiple stressors and vulnerability factors, i.e., how family income affects child depression through parental involvement and children’s self-esteem and the differences between single-parent families and intact families.

### 1.1. The Relationship between Family Income and Child Depression

Based on the vulnerability–stress model, socioeconomic factors reflecting stressful living circumstances have also been implicated in stress [14]. As the core component of family factors, the level of family income reflects the necessary material and educational resources that parents can provide for children’s physical development, psychological development, and social adaptation. Extant studies have found that a low family income is a chronic stressor affecting depression [6]. Compared with those in high-income families, people in low-income families are more likely to suffer from depression [15]. Even when the family income has been reduced but not to a low level, the proportion of those affected by depression increases [16]. Family income is an important constituent indicator of socioeconomic status [17]. Studies have shown that the lower a family’s socioeconomic status is, the higher the incidence of depression in children is [18]. Additionally, the family’s socioeconomic status in early life can predict individual’s later depressive symptoms [19]. Based on the above, our first hypothesis is that family income is negatively associated with depression in children.

To explore how family income affects depression for further intervention, most studies focused on three aspects: family environmental factors [20], personal psychological factors [21], and biological factors [22]. Family income is a distal factor affecting depression, but previous studies often focused on the impact mechanism of a single aspect [23]. Therefore, this paper intended to explore the multiple effects of family environment and personal psychology on the influence of family income on depression.

### 1.2. The Chain Mediating Effect of Parental Involvement and Children’s Self-Esteem

Family income can affect children’s development by influencing parental investment, which includes money, time, and support [24]. Previous studies have shown that a low income increases pressures on parents, which in turn limits the resources, such as materials and time, that parents can provide for their children [25]. Parental involvement is one aspect of positive parenting behavior, which mainly refers to the degree of parents’ understanding, interest, and willingness to participate in children’s daily activities. It also reflects the companionship, energy, and support invested by parents in the process of raising children. Prior studies have demonstrated that in low-income families, parents typically exhibit negative parenting behaviors, including inadequate parental involvement [26,27], which in turn is detrimental to children’s development [28]. In contrast, positive parental involvement can alleviate depressive symptoms and reduce the risk of depression in children [9]. In addition, parental involvement longitudinally predicts the development of children. Individuals with high parental involvement in childhood have a 30% to 40% decreased proportion of depression in adulthood [29]. In summary, the second hypothesis is that parental involvement serves as a mediator between family income and depression in children. Specifically, a low family income is related to negative parental involvement and a subsequent increase in depression.

Self-esteem is an assessment of one’s self-worth [30] and is a core trait that is closely linked with mental health [31]. The vulnerability–stress model considers low self-esteem a risk and persistent vulnerability factor for depression. Studies have shown that family income positively predicts students’ self-esteem [32]. Children in high-income families have relatively high levels of self-esteem [33]; even in college, individuals from high-income families exhibited higher self-esteem than those from low-income families [34]. It has also been found that self-esteem can have a negative impact on depression [35]. Based on this, the third hypothesis is that self-esteem serves as a mediator between family income and children’s depression, and children from low-income families are more likely to have low self-esteem and thus suffer from depression.

The sociometer theory of self-esteem posits that self-esteem is the measure of an individual’s interpersonal relationships and rises or falls with their quality [36]. Since relationships with parents are crucial, children incorporate parental involvement into their self-concept, which in turn influences their self-esteem [37]. It has also been found that parental involvement and warmth promote children’s self-esteem [32]. A cross-lagged study showed that family income and parental involvement can predict self-esteem [38], which is related to depression [35]. Therefore, according to the vulnerability–stress model, family income as a stressor influences self-esteem by affecting low parental involvement (direct stressor) and then influences depression [39]. In summary, the fourth hypothesis proposes that parental involvement and self-esteem play a chain mediating role in the influence of family income on depression in children.

### 1.3. Differences between the Single-Parent Family and the Intact Family

The latest data released by the Pew Research Center of the United States have shown that the global average proportion of single-parent families is 7%, and 23% of American minors are from single-parent families [40]. According to the report published by the Ministry of Civil Affairs of China, the divorce rate rose from 2.0% in 2010 to 3.4% in 2019 [41,42], which has led to increase in the number of children raised in single-parent families. Compared with intact families, children from single-parent families face more physical and mental health problems [15], including fewer positive psychological states [43], a higher risk of depression [9,44], and lower self-esteem [45]. These negative outcomes also continue into adulthood [46].

There is a disparity in family resources and parental behaviors between single-parent families and intact families. Compared with intact families, parents from single-parent families provide a lower socioeconomic status and fewer family resources [27] and tend to adopt negative parenting styles [47]. Parenting behaviors in different families will have different consequences too, as authoritative parenting is related to children’s self-esteem in intact families but not in single-parent families [48], and parental involvement in intact families is more influential on children’s self-esteem than it is in single-parent families [49]. As for children’s mental health, depression and other emotional adjustment problems are closely associated with dysfunctional family systems, which are often caused by family stress from single-parent families [50]. Therefore, different family structures, parental involvement, and children’s self-esteem may play different roles in the influence of family income on depression. Based on this, the fifth hypothesis was proposed: the underlying mechanisms of family income on depression are different between single-parent families and intact families.

**Figure 1:**
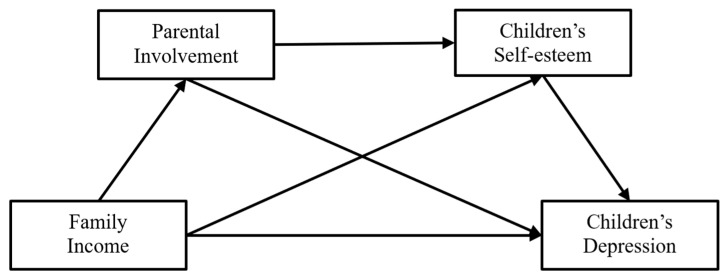
The assumed model of the chain mediating effects of parents’ involvement and children’s self-esteem on the relationship of family income with children’s depression.

In summary, based on the vulnerability–stress model of depression and previous studies, we proposed five hypotheses and constructed a mediation model (Figure 1) of the relationship between family income and depression, including the underlying mechanism of parental involvement and self-esteem.

## 2. Materials and Methods

### 2.1. Procedure and Participants

This study explored the influence of family and personal factors on children’s mental health. Children are more susceptible to family factors than adolescents [51], and a cognitive vulnerability to depression begins to develop in childhood [52]. Therefore, we selected elementary school students as the subjects.

In the present study, we collected data from a regional educational quality assessment program in a developed city in China. This program was like the Organization for Economic Cooperation and Development’s (OECD) Programme for International Student Assessment (PISA) and the International Association for the Evaluation of Educational Achievement’s (IEA) Trends in International Mathematics and Science Study (TIMSS), which select students at a specific age or grade to represent a period of school stage. Like these programs, fourth-grade students were selected to represent primary school children.

Letters of information that detailed the study’s purposes and procedures were sent to all the schools, and consent of all the participants’ parents was obtained in this program. Students in the target classes were invited to participate anonymously in the survey in class. Well-trained psychology graduate students informed all participants of the authenticity, independence, integral nature of all answers, and confidentiality of the information. It took the participants about 20 min to complete the questionnaires. Each participant completed the measures independently in a self-administered format to safeguard confidentiality. All participation was voluntary, and the data were kept completely confidential.

The sample included 1355 fourth-grade students (*M* age = 10.33, *SD* = 0.71; 47.2% girls) from Futian District in Shenzhen, of which 420 students were from single-parent families (*M* age = 10.31, *SD* = 0.76; 51.2% girls), and 935 students were from intact families (*M* age = 10.33, *SD* = 0.68; 45.5% girls). In this study, intact families are those in which the child lives with his or her biological parents, excluding reconstituted families, while single-parent families refer to families that have experienced the death of one parent or divorce. Families that experienced the death of one parent accounted for 32.0% of single-parent families.

### 2.2. Measures

#### 2.2.1. Family Income

The Family Affluence Scale (FAS II) [53], a self-reported questionnaire, is significantly correlated with family income and can also measure family wealth within a country and between countries [54]. The pre-survey found that fourth graders were not able to accurately report their family income. Therefore, with reference to the existing literature [55,56,57], we adopted the commonly used family affluence scale to indicate family income status. The FAS II includes four items: (1) number of cars, (2) whether the participant has his or her own bedroom, (3) number of vacations, and (4) number of computers. Participants rated the first item (car) on a three-point scale (0 = No, 1 = Yes, 2 = two or more), the second item (own room) on a two-point scale (0 = No, 1 = Yes), and the third and the fourth items (vacations and computers) on a four-point scale (0 = No, 1 = one, 2 = two, 3 = more than two). The sum score of these 4 items is calculated, and the score range is 0–9 points, with higher scores suggesting a higher family economic status. Regarding the reliability, although the internal correlations between the FAS II items in our study were low, all items were intercorrelated (*r* = 0.15–0.30, *p* < 0.01), and Cronbach’s alpha was relatively low (0.49), which is similar to previous studies [58,59].

#### 2.2.2. Parental Involvement

Parental involvement was assessed by asking the participants whether their parents provided company for them or discussed topics with them in everyday life. This is an eight-item scale rated on a five-point scale (1 = Absolutely not to 5 = 5–7 times a week), adapted from the PISA and TIMSS (e.g., “My parents discuss my school with me” “My parents accompany me to exercise”), which could both be adopted to assess children and teenagers of different cultures. The total average score of these 8 items is calculated, and the score range is 1–5 points, with higher scores suggesting more parental involvement. The internal consistency (Cronbach’s alpha) in our samples was 0.84. The measurement model showed a good fit (χ^2^ [19] = 136.752, *p* < 0.001; CFI = 0.964, RMSEA = 0.068, SRMR = 0.029).

#### 2.2.3. Self-Esteem

Self-esteem was assessed using the Self-Esteem Scale (SESR) [60,61]. This is a ten-item scale rated on a four-point scale (1 = strongly disagree to 4 = strongly agree). The average score of these 10 items (after negative scoring) is calculated, and the score range is 1–4 points (e.g., “I can do things as well as most people” “I take a positive attitude toward myself”), with higher scores suggesting higher levels of self-esteem. The SESR had only one dimension that could be applied to different cultures [62]. In previous research, Cronbach’s alpha of SESR was 0.77 in children [63]. The internal consistency (Cronbach’s alpha) in our samples was 0.79. The measurement model showed a good fit (χ^2^ [34] = 178.246, *p* < 0.001; CFI = 0.954, RMSEA = 0.056, SRMR = 0.036).

#### 2.2.4. Child Depression

The Children’s Depression Inventory-Short Version (CDI–S) [64] was used to assess depressive symptoms in our participants. The CDI-S is a ten-item scale. Each item measures the frequency of one depressive symptom, which is rated as 0, 1, and 2 (e.g., sadness: “I am sometimes sad”, “I am often sad”, or “I am sad all the time”). The sum score of these 10 items (after negative scoring) is calculated, and the score range is 0–20 points, with higher scores suggesting more depressive symptoms over the past two weeks. The CDI-S has been widely used around the world to measure depressive symptoms in children, and Cronbach’s alpha for the CDI-S was 0.80 in prior research [65]. The internal consistency (Cronbach’s alpha) in our samples was 0.84. The measurement model showed a good fit (χ^2^ [35] = 271.586, *p* < 0.001; CFI = 0.934, RMSEA = 0.071, SRMR = 0.037).

### 2.3. Statistical Analysis

In this study, we first performed descriptive statistics (means and standard deviations), conducted analyses of variance, and examined the group differences between single-parent families and intact families using independent sample t test, with Cohen’s d being the indicator of effect size. Pearson correlations between the key variables were analyzed too. We then used Model 6 of the PROCESS macro [66] to test the chain mediation in the total sample and in the two subgroups separately, with 5000 bootstrapping resamples and bias-corrected 95% confidence intervals. PROCESS is an SPSS macro that was specifically developed for testing complex models and has been widely used to test chain mediating models. Statistical significance was set at *p* < 0.05 (two-tailed). All of the above statistical analyses were conducted using SPSS Statistics 26. In addition, the validity of the measures was assessed using Mplus Version 8.3, including the significance level of the chi-square statistic (non-significant), the comparative fit index (CFI), the root means square error of approximation (RMSEA), and the standardized root mean square residual (SRMR).

## 3. Results

### 3.1. Preliminary Analyses

The means, SDs, chi-squared test, independent samples *t* test of the demographic, and key variables by family structures are presented in Table 1. Children from single-parent families were more likely to experience lower parental involvement (*t* = 4.66, *p* < 0.001), lower self-esteem (*t* = 3.70, *p* < 0.001), and higher depression (*t* = −5.24, *p* < 0.001) than children from intact families, and the effect sizes were all small. In addition, there was no significant difference between single-parent families and intact families in family income (*t* = 1.55, *p* = 0.121), and the effect size was tiny.

The correlation analysis results among the key variables are shown in Table 2. In both single-parent and intact families, family income was positively related to parental involvement (single-parent families: *r* = 0.24, *p* < 0.001; intact families: *r* = 0.19, *p* < 0.001) and children’s self-esteem (single-parent families: *r* = 0.15, *p* < 0.01; intact families: *r* = 0.18, *p* < 0.001), parental involvement was positively related to children’s self-esteem (single-parent families: *r* = 0.28, *p* < 0.001; intact families: *r* = 0.29, *p* < 0.001), and child depression was negatively related to parental involvement (single-parent families: *r* = −0.21, *p* < 0.001; intact families: *r* = −0.32, *p* < 0.001) and children’s self-esteem (single-parent families: *r* = −0.61, *p* < 0.01; intact families: *r* = −0.60, *p* < 0.01). In addition, family income was only significantly related to child depression in intact families (*r* = −0.09, *p* < 0.01).

### 3.2. The Mediating Role of Parental Involvement and Child Depression

**Figure 2:**
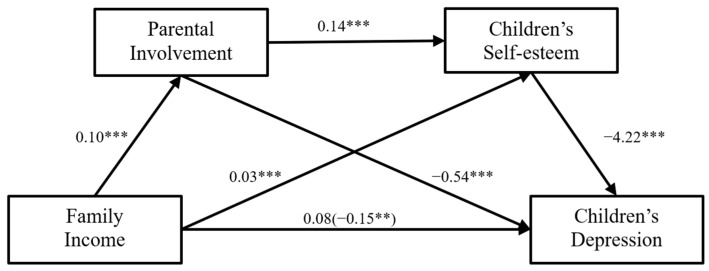
The association between family income and children’s depression with each pathway in the multiple mediation model. ** *p* < 0.01, *** *p* < 0.001.

The chain mediating model of parental involvement and self-esteem in the influence of family income on child depression is shown in Figure 2. The results indicated that the total effect of family income on child depression was significant (*β* = −0.15, *p* < 0.01), supporting our first hypothesis. Furthermore, the direct effects of family income on parental involvement (*β* = 0.10, *p* < 0.001) and children’s self-esteem (*β* = 0.03, *p* < 0.001) were significant, as was the direct effect of parental involvement on children’s self-esteem (*β* = 0.14, *p* < 0.001). The results also revealed that the direct effects of parental involvement (*β* = −0.54, *p* < 0.001) and children’s self-esteem (*β* = −4.22, *p* < 0.001) on depression were significant. Meanwhile, the residual direct effect of family income on child depression was not significant (*β* = 0.08, *p* = 0.06). Therefore, parental involvement and children’s self-esteem fully mediated the relationship between family income and child depression.

In Table 3, all three indirect effects of family income on child depression are shown. The mediating effects of parental involvement (bootstrap estimate = −0.054, 95% CI = −0.080, −0.032), children’s self-esteem (bootstrap estimate = −0.117, 95% CI = −0.178, −0.058), and the chain mediating effect of parental involvement and children’s self-esteem were all significant (bootstrap estimate = −0.060, 95% CI = −0.083, −0.040), which supports our second, third, and fourth hypothesis.

### 3.3. The Mediating Role of Parental Involvement and Child Depression in Different Family Structures

**Figure 3:**
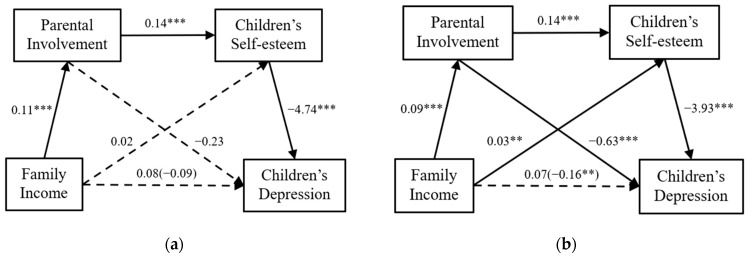
The chain mediation models in (**a**) single-parent family groups and (**b**) intact family groups. ** *p* < 0.01, *** *p* < 0.001.

We tested the chain-mediated models in single-parent family groups and intact family groups. The results are shown in Figure 3.

In single-parent families, the effects of family income on parental involvement (*β* = 0.11, *p* < 0.001), parental involvement on children’s self-esteem (*β* = 0.14, *p* < 0.001), and children’s self-esteem on child depression (*β* = −4.74, *p* < 0.001) were all significant. Furthermore, the residual direct effects of family income on child depression (*β* = 0.08, *p* = 0.32), family income on children’s self-esteem (*β* = 0.02, *p* = 0.24), and parental involvement on child depression (*β* = −0.23, *p* = 0.22) were not significant, and the total effect of family income on child depression was also not significant (*β* = −0.09, *p* = 0.40).

In intact families, the effects of family income on parental involvement (*β* = 0.09, *p* < 0.001), parental involvement on children’s self-esteem (*β* = 0.14, *p* < 0.001), and children’s self-esteem on child depression (*β* = −3.93, *p* < 0.001) were also significant. Meanwhile, the direct effect of family income on child depression (*β* = 0.07, *p* = 0.13) was not significant, but the direct effects of family income on children’s self-esteem (*β* = 0.03, *p* < 0.001) and parental involvement on child depression (*β* = −0.63, *p* <0.001) were significant. In addition, the total effect of family income on child depression was also significant (*β* = −0.16, *p* < 0.01).

Table 3 shows that the mediation of parental involvement (bootstrap estimate = −0.026, 95% CI = −0.076, 0.017) and children’s self-esteem (bootstrap estimate = −0.074, 95% CI = −0.200, 0.050) were not significant in single-parent families; meanwhile, the chain mediation of parental involvement and children’s self-esteem were significant (bootstrap estimate = −0.074, 95% CI = −0.127, −0.033). In intact families, the mediation of parental involvement (bootstrap estimate = −0.056, 95% CI = −0.086, −0.031), children’s self-esteem (bootstrap estimate = −0.127, 95% CI = −0.198, −0.060), and the chain mediation of parental involvement and children’s self-esteem were all significant (bootstrap estimate = −0.050, 95% CI = −0.075, −0.029). In summary, the impact of family income on depression was different between single-parent families and intact families, supporting our fifth hypothesis.

## 4. Discussion

The current study examined the relationship between family income and child depression, as well as the underlying mechanism of parental involvement and children’s self-esteem. We found that family income can negatively affect child depression both independently and accumulatively (i.e., chain mediation) by parental involvement and children’s self-esteem.

Our finding reconfirms the vulnerability–stress model, which states that a low family income as a family-related stressor is often related to a poor family environment (e.g., less parental involvement), and combined with vulnerability factors (e.g., low self-esteem), this can lead to an increase in depression. Consistent with previous research, family income does not exhibit a direct association with children’s developmental outcomes [67]. Instead, family income is more predisposed to influence the individual’s experience by affecting other family factors, which will then affect the psychological development [68]. Similarly, a study conducted among Chinese adolescents also found that social support and optimism had a serial mediating effect on the relations between family SES and depression, which highlights the significance of social factors and individual vulnerability [69]. In domestic situations, parents without survival pressure will invest more in their child, and such parental involvement in terms of time and energy has a crucial impact on children’s psychological development [70]. The support gained from parents can help children build confidence and socialize with others, thus contributing to their enhanced emotional functioning.

Meanwhile, we also found that the mechanism of the influence of family income on depression is different between single-parent families and intact families. In our study, family income affects depression only through the chain mediation of parental involvement and self-esteem in single-parent families, but the independent mediating effect of parental participation and self-esteem are also significant in intact families.

One fundamental reason for this variation could stem from the absence of certain family members and the reduction in support in single-parent families, which ultimately diminishes family resilience [71], which may lead to a more restricted and less adaptable pattern of interaction among members within families. Family resilience refers to the dynamic process through which a family effectively utilizes its inherent advantages and resources, collaboratively withstands pressure and challenges, and achieves optimal family adaptation and long-term development [72]. If the family income is low, single parents may face greater economic pressure and be unable to provide sufficient material and emotional support, so that family cooperation is blocked, family resilience is reduced, and the parent–child interaction is characterized by high severity and low warmth [7], thus affecting children’s self-esteem and increasing their risk of depression. For those in intact families, on the other hand, who have not experienced family disintegration, the role of family income is more pronounced and direct. Additionally, according to sociometer theory [30,36], self-esteem can be used as an indicator of the interpersonal environment that is experienced: the more valued by the people around them an individual is, the higher his or her self-esteem is. The self-esteem of children in single-parent families is affected by one parent, but it is affected by both parents in intact families.

Single-parent families themselves are more likely to face more risk factors, such as parenting stress, stress from a divorce or the death of a partner, and financial stress. When multiple risk factors coexist in the environment, they tend to act in a cumulative manner and have a negative impact on child development [10], which may greatly reduce the positive effects of household income but can be buffered by sufficient support and love of the present parent. However, the relationship between parental involvement and children’s depression in single-parent families can be complex, and hence, no significant link was revealed in single-parent families. Generally, parents in single-parent families devote more to their children, but such close connection may not always be beneficial to the child’s development. When getting along with their children, parents in single-parent families are inclined to show more emotional expressions, especially excessive criticism of their children [73]. This could be a stressor that has a greater impact on children’s depression, especially for those with lower self-esteem [74]. Our study has also shown that children’s self-esteem in single-parent families is significantly lower than in intact families. Therefore, in some cases, parental involvement does not necessarily reduce children’s risk of depression, bringing about mixed results.

Nonetheless, the complete chain-mediated effect of family income on depression is significant in both single-parent families and intact families, suggesting that even with a low family income, high parental involvement still reduces child depression by improving their self-esteem. Compared to prosperous living conditions, the appropriate participation and companionship of parents may be more influential in reducing children’s depressive symptoms. Therefore, in single-parent families, parents should not only focus on the material conditions that they can provide for their children but help to improve children’s self-esteem and ameliorate child depression by increasing parental involvement, especially support and warmth. In intact families, meanwhile, parental involvement is also crucial, so parents should guide their children to build self-esteem, educate them to have a correct understanding of themselves, and pay more attention to their inner feelings, especially in low-income families.

The present study had several limitations that are important to acknowledge. First, this study is cross-sectional. When interpreting results, causality should be carefully considered. Future research could confirm the causal relationship between these variables through longitudinal or experimental studies. Second, we measured objective family income in this study; however, subjective feelings about family income may have a greater impact on depression in children. Additionally, Cronbach’s alpha of the FAS II was low (0.49) in our study, and the items may not be very applicable in modern society. Future research could consider the impact of subjective feelings or differences in subjective and objective family income on depression and adopt more reliable measures to reflect family income. Third, since the participants in this study were all from the same region and the same grade, the representativeness of the sample was limited to some extent, and this warrants expansion to a wider group. Despite these limitations, our findings demonstrated the associations among family income, parental involvement, self-esteem, and child depression in different family structures.

## 5. Conclusions

This study investigated the chain mediating mechanism of family income on depression in children and whether the mechanism is the same in single-parent and intact families. The results revealed that family income was negatively associated with child depression through both independent mediating and chain mediating effects of parental involvement and self-esteem. Furthermore, family income was only negatively associated with child depression through a chain mediating path in single-parent families. The group differences in the mechanism of child depression in single-parent families and intact families provide a reference for future research on depression intervention for children from different family structures.

## Figures and Tables

**Table 1:** Means, standard deviations, and independent samples *t* test among the key variables.

	Single-Parent Families		Intact Families		*t*	Cohen’s d
	*M*	*SD*	*M*	*SD*		
Age	10.31	0.76	10.33	0.68	0.61	−0.03
Gender (boy)	205	48.8	510	54.5	3.83 *	
Only one child	262	62.5	436	46.6	29.28 ***	
Education						
Father ≥ college	88	21.0	180	19.3	7.46 **	
Mother ≥ college	97	23.2	169	18.1	10.16 ***	
Family income	5.21	2.18	5.40	2.07	1.55	−0.09
Parental involvement	3.05	1.02	3.33	0.98	4.66 ***	−0.28
Children’s self-esteem	3.12	0.55	3.23	0.52	3.70 ***	−0.21
Child depression	4.74	4.31	3.43	3.68	−5.24 ***	0.33

* *p* < 0.05, ** *p* < 0.01, *** *p* < 0.001.

**Table 2:** Bivariate correlations among the study variables.

Total (*n*= 1355)		1	2	3
1	Family income			
2	Parental involvement	0.21 ***		
3	Children’s self-esteem	0.17 ***	0.30 ***	
4	Child depression	−0.09 ***	−0.30 ***	−0.61 ***
**Single-parent families (*n* = 420)**		**1**	**2**	**3**
1	Family income			
2	Parental involvement	0.24 ***		
3	Children’s self-esteem	0.15 **	0.28 ***	
4	Child depression	−0.07	−0.21 ***	−0.61 ***
**Intact families (*n* = 935)**		**1**	**2**	**3**
1	Family income			
2	Parental involvement	0.19 ***		
3	Children’s self-esteem	0.18 ***	0.29 ***	
4	Child depression	−0.09 **	−0.32 ***	−0.60 ***

** correlation is significant at the level of 0.01 (two-tailed). *** correlation is significant at the level of 0.001 (two-tailed).

**Table 3:** Testing the mediating effects of family income on child depression.

Family Type	Model Pathways	Estimated	95% CI	
			Lower	Upper
All Families				
	FI → PI → CD	−0.054	−0.080	−0.032
	FI → CS → CD	−0.117	−0.178	−0.058
	FI → PI → CS → CD	−0.060	−0.083	−0.040
Single-parent Family				
	FI → PI → CD	−0.026	−0.076	0.017
	FI → CS → CD	−0.074	−0.200	0.050
	FI → PI → CS → CD	−0.074	−0.127	−0.033
Intact Family				
	FI → PI → CD	−0.056	−0.086	−0.031
	FI → CS → CD	−0.127	−0.198	−0.060
	FI → PI → CS → CD	−0.050	−0.075	−0.029

FI = family income; PI = parental involvement; CS = children’s self-esteem; CD = child depression.

## Data Availability

Data will be made available upon request to the corresponding authors.
